# Dynamics of Epiretinal Membrane Peeling under Perfluorocarbon Liquid Evaluated by Intraoperative OCT

**DOI:** 10.3390/life13020253

**Published:** 2023-01-17

**Authors:** Tomaso Caporossi, Stefano Maria Picardi, Gloria Gambini, Antonio Baldascino, Matteo Mario Carlà, Andrea Molle, Alessandra Scampoli, Lorenzo Governatori, Stanislao Rizzo

**Affiliations:** 1 Vitreoretinal Surgery Unit, Fatebenefratelli Isola Tiberina Gemelli Isola Hospital, 00186 Rome, Italy; 2 Ophthalmology Unit, Catholic University Sacro Cuore, 20123 Rome, Italy; 3 Ophthalmology Unit, Fondazione Policlinico Universitario A. Gemelli IRCCS, 00168 Rome, Italy; 4 Consiglio Nazionale delle Ricerche, Istituto di Neuroscienze, 56124 Pisa, Italy

**Keywords:** epiretinal membrane, intraoperative OCT, macular surgery, perfluorocarbon liquid, dissociated optic nerve fiber layer

## Abstract

Background: The aim of this study is to provide intraoperative data demonstrating a significant difference in the membrane peeling dynamics performed under a perfluorocarbon (PFCL) bubble, compared to normal balanced saline solution (BSS). Methods: This is a prospective, interventional, single-center study on a series of 36 consecutive eyes of 36 patients affected by primary epiretinal membrane (ERM). Eighteen eyes underwent standard ERM peeling, while 18 eyes received a PFCL-assisted procedure. Intraoperative optical coherence tomography (iOCT) B-Scans were collected to evaluate the displacement angle (DA) between the underlying retinal plane and the flap of epiretinal tissue, along with the number of times the surgeon had to grab the flap during the intervention. Follow-up visits were carried out at postoperative week 1 and months 1, 3 and 6. Results: The mean DA was 164.8° ± 4.0 in the PFCL-assisted group and 119.7° ± 8.7 in the standard group, with a statistically significant difference between groups (*p* < 0.001). Moreover, we found a significant difference in the amount of ERM grabs between the two groups (7.2 ± 2.5 in the PFCL-assisted group vs. 10.3 ± 3.1 in the standard group, *p* = 0.005). The mean BCVA and metamorphopsia significantly improved in both groups (*p* < 0.05), with no significant intergroup difference at all follow-up visits. Similarly, CST significantly decreased in both groups, and final CST was similar between the two groups (*p* = 0.719). Overall, three eyes in the standard group developed postoperative dissociated optic nerve fiber layer (DONFL, 16.6%), compared to none of the PFCL-assisted group. Conclusion: We reported a statistically significant difference in the intraoperative peeling dynamics of the PFCL-assisted group, accounting for a decreased tendency in the tearing of the ERM flap and possibly reduced damage to the fiber layer, with equal effectiveness in improving visual function and foveal thickness.

## 1. Introduction

Idiopathic macular epiretinal membranes (ERMs) grow over the retinal surface through a pathogenetic mechanism that is still not completely understood, causing metamorphopsia and visual acuity reduction. As the membrane develops it progressively induces detrimental changes to the normal macular anatomy, including increased retinal thickness, ectopic inner foveal layers (EIFLs), foveal detachment, acquired vitelliform lesions, ellipsoid zone (EZ) disruption, macular edema and the distortion of blood vessels [1].

The treatment of ERM is surgical and aimed at their removal through pars plana vitrectomy. The procedure is usually guided by the injection of vital dyes and non-soluble corticosteroids (chromovitrectomy) and can be safely associated with ILM removal to reduce recurrences and more efficiently flatten retinal folds [2]. Recent works have highlighted that ILM peeling in macular surgery may determine bundle defects in the nerve fiber layer. Tadayoni et al. identified a new OCT finding of dissociated optic nerve fibers layer (DONFL), which appeared as shallow dimples in the bundle of the optic nerve fiber layer [3,4,5]. In a recent report, Park et al. detected the presence of DONFL in 10.1% of cases after ERM surgery [6].

When approaching complicated scenarios of macular surgery, the injection of perfluorocarbon liquid (PFCL) provides posterior counterpressure on the retinal surface and its use has been proved beneficial. It was employed to assist in the construction of an inverted ILM flap while simultaneously reducing the tractions on a detached retina, to assist in the stabilization of the flap and to even make it adhere onto itself to create complex multilayered patterns [7,8,9]. When performing membrane peeling maneuvers under a PFCL bubble, the surgeon is facilitated by the lower tendency of the ERM/ILM flap to tear and break and a more easily controlled end efficient peeling motion. The aforementioned behaviors could be attributed to differences in peeling dynamics provided by the counterpressure weight that the PFCL bubble exerts over the flap [10].

**Figure 1:**
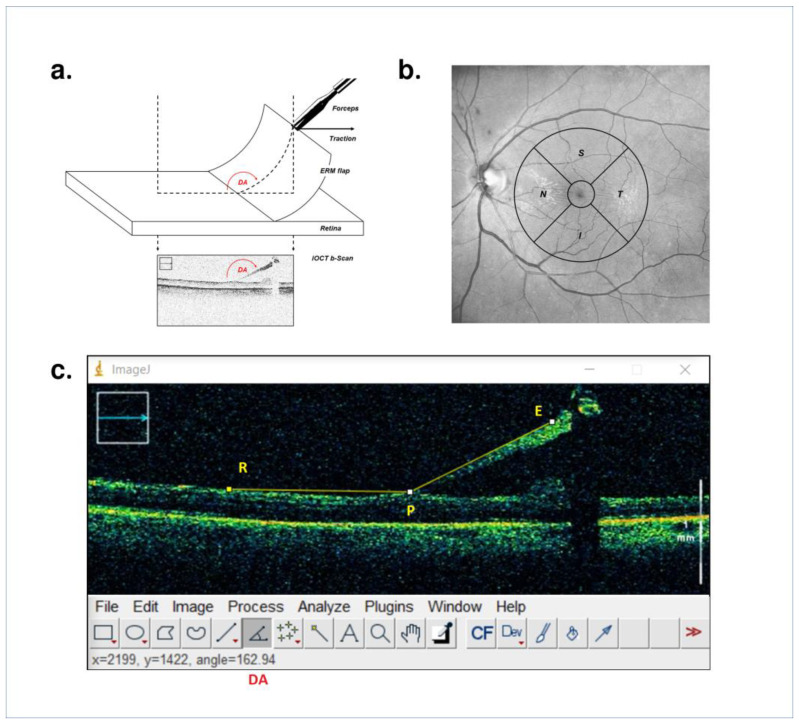
(**a**) Schematic representation of the imaging procedure, the iOCT B-Scan is oriented in the same direction as the tractional force exerted on the epiretinal membrane flap (DA = Deviation Angle). (**b**) Schematic representation of the macular subfield (S = Superior, T = Temporal, I = Inferior, N = Nasal), the central subfield represents the 1 mm-wide foveal region. (**c**) iOCT B-Scan with labels (arrowheads) and measurements (arrow) (E = Epiretinal membrane flap, P = Attachment point, R = Retina, DA = Deviation Angle).

In a previous software simulation by Dogramachi et al., a parameter named ‘Displacement Angle’ (DA—Figure 1c), which replicates the angle between an imaginary line running parallel to the retinal surface and the vector of displacement of a peeled epiretinal membrane flap, was correlated with the maximum shear stress (MSS) exerted onto the retina (R—Figure 1c), onto the point of attachment between the epiretinal membrane flap and the retinal surface (P—Figure 1c) and onto the flap itself (E—Figure 1c) [11]. To account for consistency, the simulation was designed to test for different relative stiffness values between the three elements. The study found that a favorable MSS(P)/MSS(E) ratio for efficient membrane peeling (increasing the MSS applied on the attachment points) without tearing the flap (lowering the MSS applied to the flap itself) was always obtained in a positive linear correlation trend when considering DA values above 105°. That is, the more oblique the peeling angle, the easier the removal and the less likely the tearing of the membrane flap. The study also found the MSS exerted onto the retina to be negligible for any DA, with the premise of the simulation being conducted on a model of adherent retina. A total of 10 different DA values were tested, with the widest DA of 165° being identified as optimal for safe and efficient peeling.

In a later study, Okamoto et al. observed how a wider DA could be obtained by simply grasping the membrane flap closer to the retinal surface, although this method would be rather unsafe and could increase the risk of accidental wounds. The authors further hypothesized that the counterweight of a PFCL bubble could also force a peeled membrane downwards thus safely increasing the DA without the need to adjust the forceps position [12].

The main outcome of our study is to provide intraoperative data demonstrating whether the membrane peeling maneuvers performed under a PFCL bubble can in fact result in a significantly wider DA compared to the aqueous method, by using intraoperative OCT (iOCT), a technology fully integrated into the surgical microscope and capable of providing real-time OCT images to vitreoretinal surgeons.

As secondary outcomes, we aimed to test for significant differences in postoperative morphological, structural and functional results between standard and PFCL-assisted ERM peeling in a 6 month period.

## 2. Materials and Methods

### 2.1. Population and Study Design

The study was a prospective, controlled, interventional, single-center study of a consecutive series of 36 eyes of 36 patients affected by primary ERM conducted at the Department of Ophthalmology, Catholic University of Sacred-Heart Foundation Policlinico Universitario A. Gemelli, Rome, Italy.

Inclusion criteria were age 18 or older, a clinical and instrumental diagnosis of a primary ERM at Stage 2–3 according to the OCT staging system proposed by Govetto et al. and preoperative pseudophakia. Exclusion criteria included previous ocular surgery other than uneventful cataract extraction, secondary ERM and concomitant ocular or systemic diseases that could affect BCVA and/or central subfield thickness (CST) [13].

All patients underwent comprehensive ophthalmic evaluation including best corrected LogMAR visual acuity measurement, metamorphopsia numerical evaluation using M-CHARTS score (vertical and horizontal, MV and MH), dilated anterior and posterior segment examination followed by Spectral Domain OCT (SD-OCT) volumetric scans of the macula (Solix Full-Range OCT, Optovue Inc, Freemont, CA, USA).

Prior to being scheduled for intervention, enrolled patients were evenly and randomly assigned to standard epiretinal membrane peeling (standard group, 18 eyes) and PFCL-assisted epiretinal membrane peeling (PFCL-assisted group, 18 eyes).

Follow-up visits including BCVA evaluation and M-CHARTS test were carried out at postoperative week 1 and months 1, 3 and 6. Moreover, SD-OCT scans were performed 3 and 6 months after surgery. In all patients, no other ophthalmological treatments were performed during the follow-up period.

All clinical procedures were conducted according to the tenets of the Declaration of Helsinki. Written informed consent was obtained from all patients prior to enrollment.

### 2.2. Surgical Technique

All interventions were performed by two experienced vitreoretinal surgeons (T.C., A.B.) following local anesthesia (peribulbar block) and antisepsis with povidone–iodine solution. Both groups received a standard 25-Gauge three-port vitrectomy (Constellation^®^ Vision System, Alcon Laboratories Inc, Fort Worth, TX, USA) followed by a staining of the epiretinal tissues by the injection of a combination 0.15% TrypanBlue, 0.025% Brilliant Blue G and 4% polyethylene glycol vital dye (Membraneblue-Dual^®^, DORC International, Zuidland, The Netherlands). Prior to epiretinal membrane peeling, a small bubble of approximately 1.0 cc of Perfluoro-N-Octane (EFTIAR Octane^®^, DORC International, Zuidland, The Netherlands) was injected over the macula in the PFCL-assisted group.

**Figure 2:**
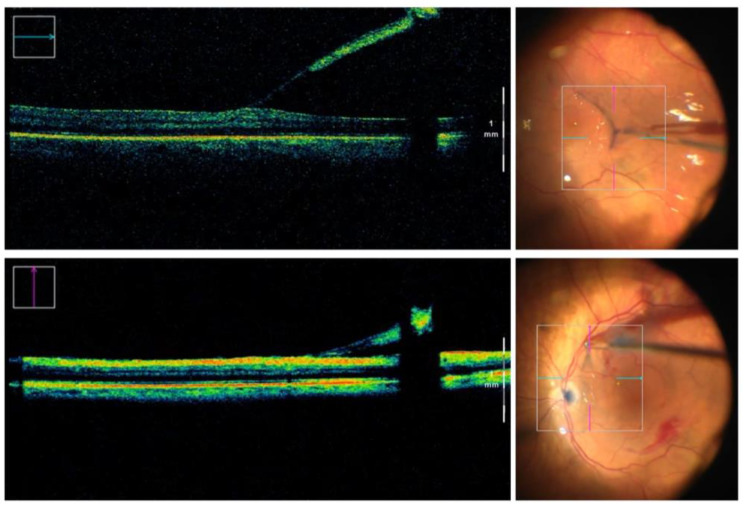
Montage of intraoperative OCT B-Scans (**left pictures**) and corresponding surgical microscope view with the superimposed crosshair lines. Two different epiretinal membrane flaps were imaged, during removal under PFCL, located at the temporal (**upper row**) and superior (**bottom row**) macular subfield.

During the intervention, upon creating a stable flap of epiretinal tissue with end-gripping forceps, the surgeon continued applying a mild traction and proceeded to acquire an iOCT B-Scan (Rescan 700^®^, Carl Zeiss Meditec AG, Jena, Germany) oriented in parallel to the projection of the vector of traction over the retina (Figure 1a). The acquired scans had a width of 6 mm and an A-Scan depth of 5.8 mm in tissue. The same imaging procedure was repeated whenever the flap was dragged into a different macular subfield or a new flap was started (Figure 2). Superior, nasal, inferior and temporal macular subfields were estimated following a simplified version of the ETDRS grid (Figure 1b). The careful evaluation of intraoperative complications such as the occurrence of retinal tears was conducted. Moreover, an independent observer (G.G.) collected intraoperative data regarding the number of grabs the surgeon had to perform with the forceps in order to complete the ERM removal.

Following a complete peeling of the ERM, the surgeon further proceeded to completely remove any residual ILM, with additional injections of vital dye as needed. In the PFCL-assisted group, the PFCL was then thoroughly removed by active aspiration and fluid/air exchange was performed in both groups.

**Figure 3:**
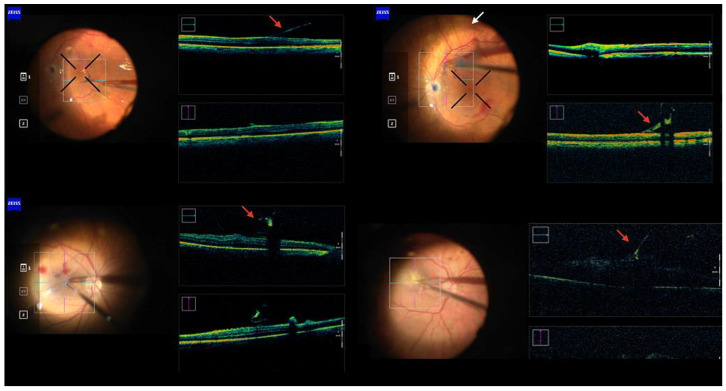
Montage of intraoperative OCT B-Scans during PFCL-assisted membrane peelings (**upper row**) and during standard peelings (**lower row**). The red arrows indicate the membrane flap. The white arrow indicates the edge of the PFCL bubble.

All OCT images for each eye were exported to external software (ImageJ, NIH, USA; version 1.53e) for postoperative analysis (Figure 1c). Two masked independent ophthalmologists (S.M.P., G.G.) assessed the DA value for individual OCT scans (Figure 3) and then reported the mean value expressed in degrees for each eye for statistical analysis. A minimum of four scans—at least one for each macular subfield—per eye were examined. The interclass correlation coefficient (ICC) was determined to assess interobserver agreement. The statistical analysis of the results was conducted via the IBM SPSS Statistics^®^ software (version 25).

### 2.3. Statistical Analysis

The statistical analysis was conducted using STATA software version 15.1 (StataCorp. College Station, TX, USA). Our sample’s normality was determined using the Shapiro–Wilk test, and *p* > 0.05 was utilized to confirm the null hypothesis. We conducted an Analysis of Variance (ANOVA) and employed the Dunnett’s multiple comparison test to evaluate the differences among retinal parameters in different follow ups. Mean DA were compared between the two groups by Mann–Whitney test; BCVAs, M-CHARTS scores and CMTs were compared intragroup (differences between baseline and month 3–6 follow-up) by paired-samples *t*-test. For contingency analysis, Chi-square and Fisher’s exact tests were utilized. Quantitative values were expressed as mean ± SD and a *p* value < 0.05 was considered statistically significant. A designated confidence interval (CI) of 95% was used. The significance threshold was established at *p* = 0.05.

## 3. Results

Population data and baseline characteristics are reported in Table 1.

All surgical interventions were carried out successfully in both groups and no significant intraoperative or postoperative complications were observed.

The mean DA was 164.8° ± 4.04 in the PFCL-assisted group and 119.7° ± 8.7 in the standard group, with a statistically significant difference between groups (Mann–Whitney test, *p* < 0.001). There was a high interobserver agreement in the DA measurements (single measurements ICC = 0.937; average measurements ICC 0.988). Moreover, we found a significant difference in the amount of ERM grabs between the two groups (7.2 ± 2.5 in the PFCL-assisted group vs. 10.3 ± 3.1 in the standard group, *p* = 0.005).

The mean LogMAR BCVA in the PFCL-assisted group significantly improved from a baseline value of 0.38 ± 0.18 to a postoperative value of 0.32 ± 0.11 at month 3 and 0.264 ± 0.156 at month 6 (*p* < 0.05, paired *t*-test), while CST significantly decreased from a baseline value of 471 ± 78 µm to a postoperative value of 437 ± 45 µm at month 3 (*p* < 0.05, Wilcoxon signed ranks test) and 427 ± 47 µm at month 6 (*p* < 0.05, Wilcoxon signed ranks test). Furthermore, the pre-operative mean M-CHARTS scores were 1.4 ± 0.4 and 1.1 ± 0.3 for MH and MV, respectively. At 6 months’ follow up, the MH significantly reduced to 0.4 ± 0.2 (*p* = 0.001, ANOVA), while MV reduced to 0.7 ± 0.3 (*p* = 0.01, ANOVA).

The mean LogMAR BCVA in the standard group significantly improved from a baseline value of 0.20 ± 0.13 to a postoperative value of 0.16 ± 0.12 at month 3 and 0.11 ± 0.10 at month 6 (*p* < 0.05, paired *t*-test), while CST significantly decreased from a baseline value of 445 ± 85 µm to a postoperative value of 395 ± 33 µm at month 3 (*p* < 0.05, Wilcoxon signed ranks test) and 371 ± 30 µm at month 6 (*p* < 0.05, Wilcoxon signed ranks test). In this group, pre-operative average M-CHARTS scores were 1.5 ± 0.3 and 1.0 ± 0.3 for MH and MV, respectively. At the end of follow up, both MH (0.6 ± 0.3) and MV (0.8 ± 0.3) showed a significant reduction when compared to pre-operative values (*p* = 0.002 and *p* = 0.03, ANOVA).

The differences in mean LogMAR BCVA and mean CST improvements were not statistically significant between the two groups at both postoperative 3 months (*p* = 0.364 and *p* = 0.460, independent samples *t*-test) and postoperative 6 months (*p* = 0.719 and *p* = 0.197, independent samples *t*-test). Similarly, comparable metamorphopsia parameters were reported between the two groups at the end of the follow up period (*p* = 0.31, independent samples *t*-test).

Furthermore, post-operative OCT analysis showed that three eyes in the standard group developed a DONFL at both 3 and 6 months’ follow-up (16.7%). In contrast, none of the eyes of the PFCL-assisted group developed any DONFL.

A summary of the improvements in mean LogMAR BCVA and mean CST in the two groups can be found in Table 2.

## 4. Discussion

Since its first introduction, many advancements have been achieved in the field of ERM surgery, including the implementation of dyes for better visualization [14,15] and new surgical instruments for safer and more efficient removal. Additionally, further investigations are being undertaken to assess the importance of adjunctive ILM peeling [16,17]. The present study aimed to observe ERM surgery from a mechanical standpoint, evaluating the presence of significant differences in the peeling dynamics under different vitreal mediums and relating them to potential differences in structural and functional postoperative outcomes.

As expected, visual acuity in both groups significantly improved after surgery and continued to improve from postoperative month 3 to 6, while foveal thickness significantly decreased in both groups.

The analysis of intraoperative data collected between the study groups demonstrates how a significantly wider DA can be achieved by merely applying a PFCL bubble over the macula prior to ERM peeling, without drastically altering the grasping position or the conventional motions of the surgical forceps. Moreover, we found a reduction in the number of times the membrane was left and taken back with forceps in the PFCL-assisted group (7.2 times in the PFCL-assisted group compared to 10.3 times in the standard group), suggesting greater stability and a lower risk of membrane tearing. No intraoperative and postoperative complications were observed in either group and no significant intergroup differences were found in mean postoperative LogMAR BCVA and mean CST at postoperative 3 and 6 months. Similarly, we found analogous results regarding postoperative metamorphopsia, with comparable M-CHARTS values between the two groups.

In other words, while the addition of PFCL proved to be safe and well-tolerated in uncomplicated cases of macular surgery, it did not prove to be more beneficial in producing better functional or anatomical results in terms of CST. Interestingly, the latter finding seems to be in accordance with the assumption that the overall MSS exerted on the adherent retina is not influenced by the DA. Moreover, the incidence of surgical complications such as the formation of retinal breaks was equally null in both groups as the intraoperative strain exerted on the neural retina, considered as a single slab of tissue, did not differ enough between groups to cause a significant long-term disparity in postoperative macular thickness. Of note, the presence of a mild separation between the interdigitation zone in the outer retina and the retinal pigmented epithelium (RPE) that can be found in cases of ‘cotton ball sign’ or acquired vitelliform lesions could undermine the assumption of ‘adherent retina’ [18,19].

According to Dogramaci et al., a significant difference in relation to the DA value exists in the MSS exerted on the adhesion points (***p***) between the ERM and the retinal surface. Although not part of the main outcomes of our study, we indeed reported the development of DONFL in three patients in the standard group (16.7%), while none of the PFCL-assisted group developed such a complication. Recent research conducted by Park et al. showed that DONFL was visible in 10.2% of cases after epiretinal membrane surgery, and it was principally correlated with ILM peeling and intravitreal gas tamponade, but no information about the mechanics of the surgical removal of the membrane were presented [6]. Steel et al. previously hypothesized that the degree of DONFL seen after ILM peeling in macular hole surgery appeared to be influenced by the ILM peeling technique (forceps or diamond dusted membrane scraper) and perhaps other surgeon-related factors [20]. We can speculate that PFCL-assisted epiretinal membrane removal, thanks to the higher DA, may induce a more physiological and slowly progressing dissection of the epiretinal membrane, reducing the postoperative formation of DONFL in comparison with the standard technique. These findings unfortunately lack statistical meaning due to the small number of cases we analyzed, but could act as a starting point for further research focusing on DONFL as a specific biomarker of the inner retinal layers, especially in cases where there is evidence on preoperative OCT of tightly adherent ERMs [21].

Moreover, although approaching a wider DA associated with a lower shear stress applied on the flap is to be considered advantageous in itself, further considerations should be made regarding the mechanical changes in the tissue, including its elasticity and intrinsic shear stress resistance. During surgery, we found that epiretinal membrane flaps possess an increased stiffness when handled inside the PFCL bubble compared to BSS, even if such a tendency could not be objectively quantified and reported. The same tendency was also observed by Okamoto et al. in their series of 36 eyes [12].

Being hydrophobic in nature, PFCL displaces water particles outside its bubble, thus exposing polar terminal groups in proteins located over the epiretinal membrane surface. The ensuing formation of new protein links has been proposed as a potential explanation for the increased adhesiveness of ILM flaps under perfluorocarbon liquid and it could also account for the increased stiffness of the ERM by inducing a rearrangement in its molecular ultrastructure [12]. Structural changes could affect the tendency of the flap tearing regardless of the DA at which it is being peeled off the retinal surface.

The present study displays several limitations. By design, the patient’s group was not masked to the surgeons acquiring the intraoperatory images. To account for the good reliability of the measurements in a real-world scenario, surgeons strived to perform the operating maneuvers as spontaneously and safely as possible in different portions of the macula, which implies that certain factors potentially affecting the DA, including the distance of the forceps from the retinal surface and the flap length and shape, were intentionally not standardized. Owing to the small patient sample, the study was not designed to analyze the potential impact of different ERM stages or additional related OCT findings (i.e., ellipsoid zone status, EIFL, foveal detachment, acquired vitelliform lesions) on visual function and postoperative retinal thickness.

In conclusion, we were able to report a statistically significant difference in intraoperatory peeling dynamics, potentially accounting for a decreased tendency in the tearing of the epiretinal membrane flap, which was consistent with the significant reduction of ERM grabs needed to complete the peeling. The present study was conducted on ERMs because they are thicker and thus easier to capture on iOCT scans compared to the thinner flaps of isolated ILM but the same principles would apply to the latter. That is, our results reinforce the rationale of PFCL-assisted membrane peeling in procedures such as the inverted flap technique in macular holes, where flap integrity is a highly desirable factor.

We found PFCL-assisted ERM peeling to be equally safe and effective in improving visual function at up to 6 months of follow up compared to standard ERM peeling, with no significant changes in postoperative foveal thickness but possible benefits in the reduction of the postoperative insurgence of DONFL.

Further investigations, including a larger series of cases and testing for additional biomarkers, are needed to provide vitreoretinal surgeons with a comprehensive knowledge about the full spectrum of the risks and benefits of PFCL assistance in macular surgery.

## Figures and Tables

**Table 1:** Demographic data and baseline parameters in the study population divided into the two groups.

Parameters (Mean ± SD)	PFCL-Assisted Group	Standard Group	*p*-Value (Intergroup Analysis)
Number of patients	18	18	-
Age (years)	69 ± 5	68 ± 6	-
Preoperative BCVA (LogMAR)	0.38 ± 0.18	0.20 ± 0.13	-
Preoperative MH score	1.4 ± 0.4	1.5 ± 0.3	0.78
Preoperative MV score	1.1 ± 0.3	1.0 ± 0.3	0.63
Mean preoperative CST (μm)	471 ± 78	445 ± 85	0.48

BCVA = Best corrected visual acuity; MH = M-charts horizontal; MV = M-charts vertical; CST = Central subfield thickness.

**Table 2:** Intraoperative data and post-operative functional and anatomical outcomes in the two groups.

Parameters (Mean ± SD)	PFCL-Assisted Group	Standard Group	*p*-Value (Intergroup Analysis)
Intraoperative DA (degrees)	164.8 ± 4.04	119.7 ± 8.7	0.001
Number of ERM grabs	7.2 ± 2.5	10.3 ± 3.1	0.005
3 months BCVA (LogMAR)	0.32 ± 0.11	0.16 ± 0.12	0.77
3 months CST (μm)	437 ± 45	395 ± 33	0.46
6 months BCVA (LogMAR)	0.26 ± 0.15	0.11 ± 0.10	0.72
6 months CST (μm)	471 ± 78	445 ± 85	0.48
6 months MH	0.4 ± 0.2	0.6 ± 0.3	0.18
6 months MV	0.7 ± 0.3	0.8 ± 0.3	0.38

DA = displacement angle; BCVA = Best corrected visual acuity; MH = M-charts horizontal; MV = M-charts vertical; CST = Central subfield thickness. Bold characters indicate statistically significant results.

## Data Availability

The data that support the findings of this study are available from the corresponding author, S.M.P., upon reasonable request.
